# Unicortical Locking Screws Provide Comparable Rigidity to Bicortical Compression Screws in Tranverse Mid-Shaft Clavicle Fracture Plate Fixation Constructs

**DOI:** 10.3390/clinpract15060101

**Published:** 2025-05-26

**Authors:** Curtis W. Hartman, Nicholas C. Branting, Matthew A. Mormino, Timothy J. Lackner, Bradford P. Zitsch, Edward V. Fehringer, Hani Haider

**Affiliations:** Department of Orthopaedic Surgery and Rehabilitation, University of Nebraska Medical Center, Omaha, NE 68198-5640, USAtim.lackner@unmc.edu (T.J.L.);

**Keywords:** clavicle, mid-shaft fracture, biomechanical comparison, unicortical locking screws, bicortical compression screws

## Abstract

**Background**: Mid-shaft clavicle fracture fixation carries neurovascular injury risk. The purpose of this study was to compare bicortical compression and unicortical locked clavicle plate constructs biomechanically. **Materials and Methods**: Ten fourth-generation composite transverse mid-shaft clavicle osteotomy specimens were assigned to two groups, and each clavicle was fixed with an eight-hole second-generation 3.5 mm pelvic reconstruction plate placed superiorly. Group one included five fixed with bicortical compression screws and group two included five fixed with unicortical locking screws. All were tested on a four-axis servohydraulic testing frame in three modes: axial rotation, anterior/posterior bending, and cephalad/caudad bending. **Results**: Mean construct stiffness for AP bending was 1.255 ± 0.058 Nm/deg (group 1) and 1.442 ± 0.065 Nm/deg (group 2) (*p* = 0.001). Mean construct stiffness for axial rotation was 0.701 ± 0.08 Nm/deg (1) and 0.726 ± 0.03 Nm/deg (2) (*p* = 0.581). Mean construct stiffness for cephalad bending was 0.889 ± 0.064 Nm/deg (1) and 0.880 ± 0.044 Nm/deg (2) (*p* = 0.807). Mean construct stiffness for caudal bending was 2.523 ± 0.29 Nm/deg (1) and 2.774 ± 0.25 Nm/deg (2) (*p* = 0.182). **Conclusions**: With transverse mid-shaft clavicle fractures, unicortical locking fixation provided comparable rigidity to bicortical compression fixation in axial rotation, cephalad bending, and caudal bending; it provided greater rigidity in AP bending.

## 1. Introduction

The clavicle is among the more unique human bones. It serves as a strut for the upper extremity and is the only bony connection between it and the axial skeleton. Loads on the upper extremity are transmitted to the thorax through the clavicle. Combined with its superficial location, these factors make the clavicle the most fractured human bone, with a shape unlike all others [1,2].

Middle one-third clavicle fractures account for approximately 80% of clavicle fractures and have traditionally been treated non-operatively based on Neer’s work [3]. However, Hill et al. studied non-operatively treated displaced mid-shaft clavicle fracture outcomes, and found that patients with displaced fractures often fared poorly [4]. So, they recommended operative fixation for displaced mid-shaft clavicle fractures. In a randomized clinical trial, the Canadian Orthopaedic Trauma Society found that operative fixation of displaced adult mid-shaft clavicle fractures resulted in improved functional outcomes and lower rates of nonunion as well as malunion when compared with non-operative treatment [5].

Unfortunately, the orthopedic and vascular literature contains reports of both early and late limb-threatening complications associated with plate-screw constructs for mid-shaft clavicle fractures [6,7,8,9,10,11,12,13,14,15,16]. Unicortical screw fixation may reduce vascular injury risk to subclavian vessels near mid-shaft fractures as the inferior clavicular cortex is not violated with instrumentation utilizing this technique. A hybrid approach utilizing central unicortical locked screws was proposed, whose biomechanical utility was shown [17]. However, it is unclear if unicortical screws alone (and associated plate fixation) are biomechanically adequate for mid-shaft clavicle fractures. We hypothesized that 3.5 mm pelvic reconstruction plates with only unicortical screw fixation would provide comparable stability to more traditional bicortical compression screw constructs with the same plate type in a transverse mid-shaft fracture model.

## 2. Materials and Methods

Ten fourth-generation composite clavicle models (Sawbones, Pacific Research Laboratories, Vashon, WA, USA) were used in this study. Each of these synthetic clavicles was designed to replicate the properties of real human bone and had a manufactured mid-shaft osteotomy, meaning an artificial fracture was introduced at the middle section of the bone. These clavicle specimens were randomly assigned to one of two experimental groups for further study. In many biomechanical fracture studies, cadaveric specimens are used. However, bone density, quality, and size can differ significantly between specimens. We wished to change only one variable with our study: screw type.

**Figure 1:**
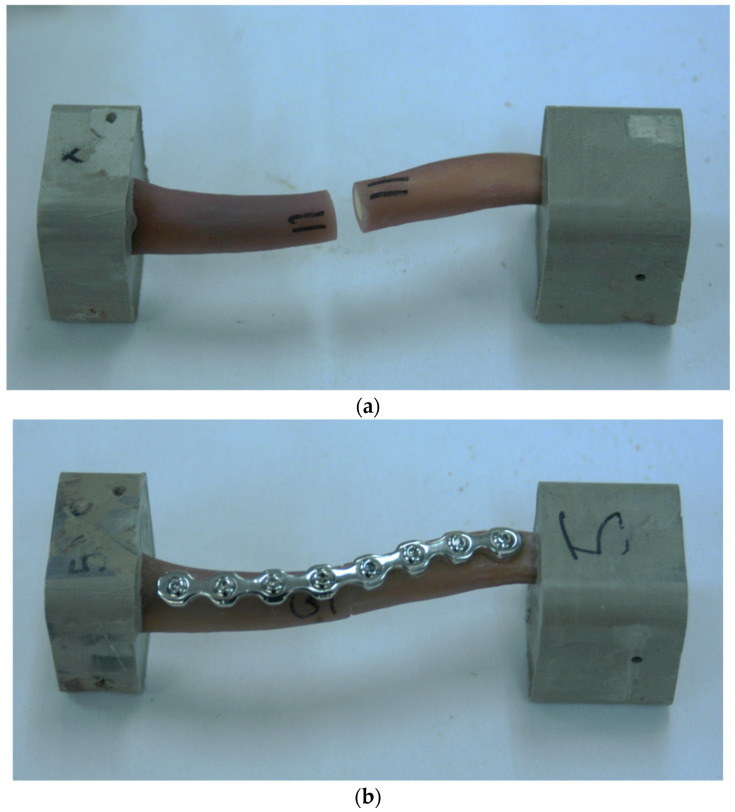
(**a**) Fourth-generation composite clavicles from Sawbones, each with a manufactured mid-shaft osteotomy; (**b**) clavicles repaired using contoured Synthes eight-hole, second-generation 3.5 mm locking pelvic reconstruction plates affixed to the superior surface of the bone.

To ensure consistency in the surgical technique, each clavicle was carefully realigned (reduced) and surgically repaired using standard orthopedic trauma procedures. A single author (CWH, a resident with four years of experience) performed all repairs under the close supervision of two experienced attending orthopedic surgeons (EVF and MAM). For each specimen, a contoured, eight-hole, second-generation 3.5 mm locking pelvic reconstruction plate (Synthes, Paoli, PA, USA) was placed on the superior surface of the clavicle to provide structural support and stabilization (Figure 1).

This study divided the specimens into two groups based on the type of screw used for fixation. In the first group, standard bicortical compression screws were used to secure the plate. These screws were tightened to a precise torque using a 1.5 Newton-meter (Nm) torque-limiting driver to ensure uniform tension and secure fixation. In the second group, unicortical locking screws were used instead, also tightened with the same 1.5 Nm torque-limiting driver. To maintain consistency, all plates were shaped in the same way using standard handheld plate benders before being affixed to the bone models. In both groups, four screws were placed on each side of the osteotomy to ensure a balanced and stable repair.

**Figure 2:**
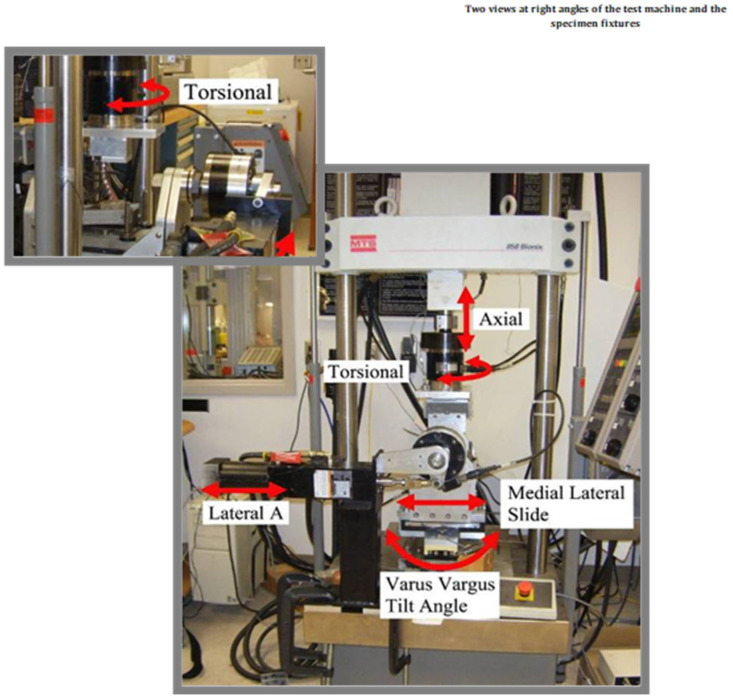
Four-axis MTS servohydraulic machine.

Once the repairs were complete, all specimens were securely embedded (potted) in liquid molding plastic to hold them in place for mechanical testing. The repaired clavicles were then subjected to stiffness testing using a four-axis MTS servohydraulic testing machine. This machine operated under displacement control and tested the specimens under three different loading conditions: axial rotation, anterior/posterior bending, and cephalad/caudad bending (Figure 2). These loading modes simulated different types of mechanical stress that clavicles may experience in real-life situations, such as twisting, forward-and-backward bending, and upward-and-downward bending.

The mechanical testing procedure included an initial warm-up loop to condition the specimens before data collection. After this, three continuous cycles of mechanical loading were performed, with data recorded at a rate of 100 Hz. The range of displacement for each test was determined by conducting a preliminary scouting trial. The final testing parameters were set as follows: axial rotation was tested with a range of ±8.0 degrees, anterior/posterior bending with a range of ±7.2 degrees, and cephalad/caudad bending with a range of +7.2 degrees to −3.6 degrees.

The first loading test conducted was anterior/posterior (AP) bending. In this test, the proximal (closer to the center of the body) end of the clavicle specimen was firmly fixed to the testing machine, while the distal (farther from the center of the body) end was placed in a custom-built movable fixture. This fixture allowed the specimen to bend naturally at the osteotomy site while minimizing unwanted shear forces. Next, the specimens were rotated 180 degrees to undergo cephalad/caudad bending testing, using the same fixture setup. Finally, the axial rotation test was performed by applying torque to the specimen through a piston actuator, simulating a twisting force on the clavicle.

To analyze the results, the collected data were grouped based on the type of screw used for fixation. Descriptive statistical analysis was performed to compare the stiffness of the two groups. A two-sample *t*-test was used to determine whether there were significant differences between the groups. The significance threshold was set at a *p*-value of ≤0.05, meaning that differences with a *p*-value at or below this level were considered statistically significant.

## 3. Results

**Figure 3:**
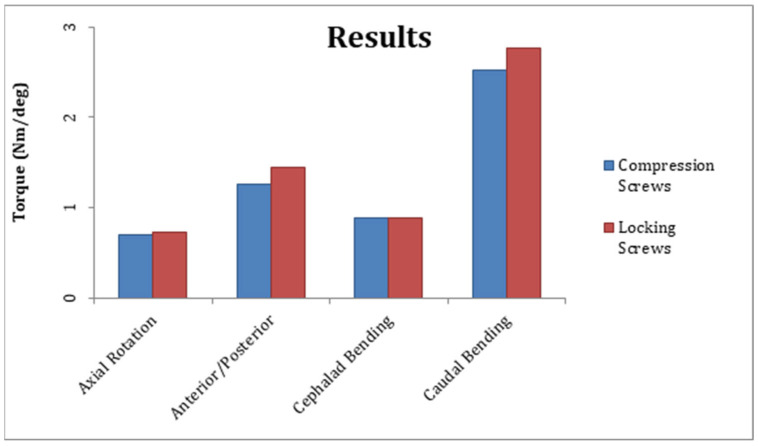
Mean steady-state construct stiffness for axial rotation, anterior/posterior bending, cephalad bending, and caudal bending between group 1 (bicortical compression screws) and group 2 (unicortical locking screws).

The mean steady-state construct stiffness for axial rotation, cephalad bending, and caudal bending was similar between groups one and two but differed significantly in AP bending (Table 1, Figure 3). The mean steady-state construct stiffness for AP bending was 1.255 ± 0.058 Nm/deg for group one and 1.442 ± 0.065 Nm/deg for group two; the difference was statistically significant (*p* = 0.0013). The mean steady-state construct stiffness for axial rotation was 0.701 ± 0.08 Nm/deg for group one and 0.726 ± 0.03 Nm/deg for group two; the difference was not significant (*p* = 0.581). The mean steady-state construct stiffness for cephalad bending was 0.889 ± 0.064 Nm/deg for group one and 0.880 ± 0.044 Nm/deg for group two; the difference was not significant (*p* = 0.807). The mean steady-state construct stiffness for caudal bending was 2.523 ± 0.29 Nm/deg for group one and 2.774 ± 0.25 Nm/deg for group two; the difference was not significant (*p* = 0.182).

## 4. Discussion

Following the Canadian Orthopaedic Society’s findings that operative fixation of displaced adult mid-shaft fractures resulted in improved functional outcomes with reduced nonunion and malunion (when compared with non-operative treatment), one assumes that clavicle fixation rates would increase [5]. Yet, clavicle fracture fixation is not without risk. Intra-operative or even post-operative vascular injury can be limb-threatening [6,7,8,9,10,11,12,13,14,15,16]. Plates and screws are familiar to orthopedic surgeons and provide excellent clavicular shaft rotational control, and constructs that do not violate the inferior clavicular cortex may allow surgical treatment while reducing vascular injury risk. Therefore, the purpose of this study was to perform a biomechanical comparison of mid-shaft clavicle fracture fixation constructs with only unicortical locked screws and those with standard bicortical compression screws.

Previously, Collinge et al. described anterior clavicular plate fixation to improve rigidity and decrease vascular injury risk [18]. But this technique requires some deltoid detachment laterally and may not allow vascular protection in all due to anatomic vascular variability. Moreover, Robertson et al. suggested anterior–inferior reconstruction plates might be subjected to loads that could cause early mechanical failure [19]. In fact, Iannotti et al. found that superior plating of mid-shaft clavicle osteotomies was biomechanically advantageous to anterior plating [20]. They also found that 3.5 mm limited-contact dynamic compression (LCDC) plates were better than 2.7 mm dynamic compression (DC) plates or 3.5 mm reconstruction plates. But it is unclear if improved stability afforded by LCDC plates is clinically important. Also of note, the Canadian group found that hardware removal incidence declined with contoured plates [5]. Finally, in a radiological study, Sinha et al. defined safe zones for drilling when fixing clavicle fractures, but Hussey et al. showed no difference in neurovascular injury risk between superior and anterior plate placement [21,22].

Mid-shaft clavicle fracture morphology may vary. Some include comminution and others include spiral components that allow interfragmentary fixation. Our simplification of this mid-shaft clavicle fracture was to allow perfect fracture reproduction in identical specimens without differences in interfragmentary fixation. Our goal was to compare standard bicortical compression screws with unicortical screws, eliminating other controllable variables. We wanted to only change one (screw type) to make it as scientific as possible. Also of note, the simple fracture line, when reduced anatomically, provides inherent stability. We chose to study the fracture construct in this fashion and for this reason eliminated the comminution issue given its infinite number of permutations. We believe the findings of this in vitro study need to be further examined in clinical trial settings in which one would utilize unicortical screws in place of bicortical compression screws in a randomized fashion to ensure baseline comparability (more specifically, transverse fracture lines or oblique fractures in which excellent interfragmentary screw fixation can be obtained).

Our study is not without limitations. We did not create a gap at the fracture site to simulate comminution. We are not of the opinion that simulated comminution and/or poor reduction are well simulated with gaps, as this biomechanical simulation can be oversimplified, given we know that the biologic environment changes continually (and with it the stability). Second, we only tested a transverse fracture model. However, oblique fracture lines frequently allow the utilization of an interfragmentary screw that can dramatically stabilize constructs and are quite variable. We felt the utilization of a transverse fracture line would be more reproducible and eliminate interfragmentary screws to allow comparison of bicortical compression and unicortical locking screws. Third, we utilized fourth-generation composite fracture models. It is possible that cadaver specimens may have changed our results. However, we wished to eliminate bone density variability as one variable that could significantly alter results, so we used consistent specimens. Fourth, we utilized second-generation pelvic reconstruction plates that may not be used by some surgeons. However, these plates also allow excellent contouring to bony surfaces. One senior surgeon (EVF) has used 3.5 mm pelvic reconstruction plates extensively for this indication as he learned from a mentor that has used them for over 30 years for both acute fractures and non-unions. The plate contours nicely versus often poorly fitting “anatomic” plating systems and LCDC and DC plates. Fifth, it is unclear if greater AP stiffness translates to improved surgical outcomes as this is beyond the scope of this study. Greater AP stiffness could potentially lead to stress shielding or other issues including peri-prosthetic fractures. Stress shielding, related to repetitive stress, could lead to weakening of the bone between screws and/or at the ends of the plate that put the bone at risk for fracture. Moreover, with increased AP bending stiffness, repetitive loading in this plane in vivo or in vitro may stress a construct so stiff that peri-prosthetic fractures occur at screw penetration sites and the ends of the plate due to hardware stress risers in these areas. Sixth, our sample size is small, five in each group. A larger sample size may have led to a more robust study, but we were limited by cost. We recognize that these limitations may not allow our results to be generalizable to all clavicle fractures or the plating systems used to address them. Yet, these limitations do not affect our ability to adequately evaluate the biomechanical differences between unicortical locked screw fixation constructs versus standard bicortical screw fixation constructs in a transverse osteotomy mid-shaft clavicle fracture model in fourth-generation composite specimens.

## 5. Clinical Relevance

Several studies have revealed good outcomes following plate/screw fixation of displaced mid-shaft clavicle fractures [4,5,18]. Catastrophic vascular injury risk mitigation with familiar fixation that does not violate inferior clavicular cortices may influence mid-shaft clavicle fracture treatment options. We believe that future studies should also include the use of smaller constructs that are more appropriate for a smaller bone such as the clavicle.

## 6. Conclusions

In a transverse mid-shaft clavicle osteotomy model, superiorly placed bicortical fixation with 3.5 mm pelvic reconstruction plates did not provide greater rigidity than similarly placed unicortical locking 3.5 mm pelvic reconstruction plate/screw fixation constructs. Unicortical locking fixation provided greater rigidity in AP bending but we are unsure of the clinical significance of this.

## Figures and Tables

**Table 1:** Mean steady-state construct stiffness for axial rotation, anterior/posterior bending, cephalad bending, and caudal bending between group 1 and group 2.

	Group 1	Group 2	*p*-Value
Axial Rotation	0.701 ± 0.08	0.726 ± 0.03	0.581
Anterior/Posterior	1.255 ± 0.058	1.442 ± 0.065	0.0013
Cephalad Bending	0.889 ± 0.064	0.880 ± 0.044	0.807
Caudal Bending	2.523 ± 0.29	2.774 ± 0.25	0.182

## Data Availability

Data is unavailable due to privacy restrictions from Synthes.

## Abbreviations

The following abbreviations are used in this manuscript:

MDPI	Multidisciplinary Digital Publishing Institute
DOAJ	Directory of open access journals
TLA	Three-letter acronym
LD	Linear dichroism

## References

[1] Post M. (1989). Current Concepts in the Treatment of Fractures of the Clavicle. Clin. Orthop. Relat. Res..

[2] Harrington M., Keller T., Seiler J., Weikert D., Moeljanto E., Schwartz H. (1993). Geometric properties and the predicted mechanical behavior of adult human clavicles. J. Biomech..

[3] Neer C.S. (1960). Nonunion of the clavicle. JAMA.

[4] Hill J.M., McGuire M.H., Crosby L.A. (1997). Closed treatment of displaced middle-third fractures of the clavicle gives poor results. J. Bone. Jt. Surg. Br..

[5] Canadian Orthopaedic Trauma Society (2007). Nonoperative treatment compared with plate fixation of displaced midshaft clavicular fractures. A multicenter, randomized clinical trial. J. Bone Jt. Surg..

[6] Johnson B., Thursby P. (1996). Subclavian artery injury caused by a screw in a clavicular compression plate. Cardiovasc. Surg..

[7] Bostman O., Manninen M., Pihlajamaki H. (1997). Complications of plate fixation in fresh displaced midclavicular fractures. J. Trauma.

[8] Shackford S.R. (2003). Taming of the screw: A case report and literature review of limb-threatening complications after plate osteosynthesis of a clavicular nonunion. J. Trauma.

[9] Kitsis C.K., Marino A.J., Krikler S.J., Birch R. (2003). Late complications following clavicular fractures and their operative management. Injury.

[10] Ding M., Hu J., Ni J., Lv H., Song D., Shu C. (2012). Iatrogenic subclavian arteriovenous fistula: Rare complication of plate osteosynthesis of a clavicle fracture. Orthopedics.

[11] Clitherow H.D., Bain G.I. (2014). Association between screw prominence and vascular complications after clavicle fixation. Int. J. Shoulder Surg..

[12] Perera K.G., Clifford C., Maddock L.J. (2015). Subclavian artery injury secondary to clavicular plate fixation: A novel operative approach. J. Surg. Case Rep..

[13] Stillwell A., Ioannou C., Daniele L., Tan S.L. (2017). Osteosynthesis for clavicle fractures: How close are we to penetration of neurovascular structures. Injury.

[14] Pallett S.J.C., Singh I., Rady N., Goshai H. (2018). Delayed Subclavian Artery Aneurysm Following Fixation of a Clavicular Fracture. Vasc. Endovasc. Surg..

[15] Lewis S.D., Chew F.S. (2019). Clavicle fixation screw impingement causing subclavian artery pseudoaneurysm. Radiol. Case Rep..

[16] Chuaychoosakoon C., Suwanno P., Boonriong T., Suwannaphisit S., Klabklay P., Parinyakhup W., Maliwankul K., Duangnumsawang Y.D., Tangtrakulwanich B. (2019). Patient Position is Related to the Risk of Neurovascular Injury in Clavicular Plating: A Cadaveric Study. Clin. Orthop. Relat. Res..

[17] Rawlings M., Knox D., Patel M., Ackland D. (2016). A hybrid approach to mid-shaft clavicle fixation. Injury.

[18] Collinge C., Devinney S., Herscovici D., DiPasquale T., Sanders R. (2006). Anterior-inferior plate fixation of middle-third fractures and non-unions of the clavicle. J. Orthop. Trauma.

[19] Robertson C., Celestre P., Mahar A., Schwartz A. (2008). Reconstruction Plates for Stabilization of Mid-Shaft Clavicle Fractures: Differences between Nonlocked and Locked Plates in Two Different Positions. J. Shoulder Elb. Surg..

[20] Iannotti M.R., Crosby L.A., Stafford P., Grayson G., Goulet R. (2002). Effects of plate location and selection on the stability of midshaft clavicle osteotomies: A biomechanical study. J. Shoulder Elb. Surg..

[21] Sinha A., Edwin J., Sreeharsha B., Bhalaik V., Brownson P. (2011). A radiological study to define safe zones for drilling during plating of clavicle fractures. J. Bone Jt. Surg. Br. Vol..

[22] Hussey M.M., Chen Y., Fajardo R.A., Dutta A.K. (2013). Analysis of neurovascular safety between superior and anterior plating techniques of clavicle fractures. J. Orthop. Trauma.

